# Repeated cross-sectional surveys show a decreasing trend in *Borrelia burgdorferi* sensu lato seroprevalence over a 50-year period, Finland, 1966 to 2017

**DOI:** 10.2807/1560-7917.ES.2025.30.36.2500171

**Published:** 2025-09-11

**Authors:** Maija Lamppu, Tero Klemola, Eero Vesterinen, Timothée Dub, Annukka Pietikäinen, Jukka Hytönen

**Affiliations:** 1 Department of Biology, University of Turku, Turku, Finland; 2 Institute of Biomedicine, Faculty of Medicine, University of Turku, Turku, Finland; 3 Department of Health Security, Finnish Institute for Health and Welfare, Helsinki, Finland; 4 TYKS Laboratories, Clinical Microbiology, Turku University Hospital, Turku, Finland

**Keywords:** seroprevalence, Lyme borreliosis, *Borrelia burgdorferi*, Finland

## Abstract

**BACKGROUND:**

Lyme borreliosis (LB) caused by *Borrelia burgdorferi* sensu lato (Bbsl) spirochetes is the most common tick-borne infection in Europe and the incidence of LB has been increasing in many countries.

**AIM:**

We examined changes in Bbsl seroprevalence in Finland over the past 50 years.

**METHODS:**

We analysed samples collected from people aged ≥ 15 years in nationwide cross-sectional health surveys conducted over the years 1966–1972, 1978–1980, 2000–2001 and 2017. Samples were screened with an IgG ELISA assay and confirmed with an IgG bead immunoassay. We assessed factors associated with Bbsl seropositivity by generalised linear models.

**RESULTS:**

Seroprevalence was highest in 1966–1972 (25.0%; 95% confidence interval (CI): 22.3–27.7%), while it was lower in 1978–1980 (16.6%; 95% CI: 14.3–18.9%), 2000–2001 (7.4%; 95% CI: 5.8–9.0%) and 2017 (3.4%; 95% CI: 2.3–4.5%). Male sex (p = 0.0014) and increasing age (p < 0.0001) were associated with higher seropositivity. The estimated probability of being seropositive was highest among residents from southern (least squares (LS) mean: 0.164; 95% CI: 0.139–0.192), central and eastern Finland (LS mean: 0.141; 95% CI: 0.116–0.170) and lowest in northern Finland (LS mean: 0.019; 95% CI: 0.014–0.028).

**CONCLUSION:**

Our results show a decrease in the seroprevalence in Finnish people over time. Reasons for this decrease are not clear but could be related to urbanisation, increased awareness, effective diagnostics and prompt antibiotic treatments. Overall, this study demonstrates how repeated serosurveys can help in revealing trends and identifying potential risk groups.

**Key public health message**

**What did you want to address in this study and why?**

Lyme borreliosis (LB), caused by *Borrelia burgdorferi* sensu lato (Bbsl) bacteria, is the most common tick-borne infection in the northern hemisphere and increasingly diagnosed. We examined how the presence of antibodies against Bbsl among Finnish adults has changed by analysing serum samples from four different nationwide health surveys conducted over a period of 50 years, from the late 1960s to 2017.

**What have we learnt from this study?**

We observed a decreasing trend in seroprevalence from 25.0% in the first survey to 3.4% in 2017. Males and older adults (aged ≥ 70 years) had more often antibodies against Bbsl than females and younger people. Moreover, a substantial regional variation was observed, although regional differences levelled off in 2017.

**What are the implications of your findings for public health?**

We do not know the reasons for this decrease but urbanisation, increased awareness of ticks, effective diagnostics and prompt antibiotic treatment could explain the decreasing trend. Continued surveillance and preventive measures, such as awareness campaigns, are essential in decreasing risks of LB and other tick-borne diseases.

## Introduction

Lyme borreliosis (LB), the most common tick-borne disease in Finland, is caused by *Borrelia burgdorferi* sensu lato (Bbsl) spirochetes. In Finland, these bacteria are transmitted to humans mainly by two tick species, *Ixodes ricinus* and *Ixodes persulcatus* [1]. The clinical presentation of LB can vary greatly depending on the stage of the disease. In the early phase of the infection, most LB patients develop erythema migrans (EM), which is an expanding skin rash near the tick attachment site. Other clinical manifestations of LB may include various forms of disseminated LB such as neuroborreliosis, arthritis and chronic skin inflammation [2]. In Europe, around half of Bbsl infections are assumed to be symptomatic, although this proportion is uncertain and likely varies between surveys and countries due to underdiagnosis and inconsistent surveillance data [3].

Although ticks and Bbsl have been around for several thousands of years and there is documented history of symptoms related to LB already from the late 19th century, Bbsl was identified as the causative agent of LB only in the early 1980s, and since the 1990s, epidemiological studies on LB have been conducted in Europe [4]. Today, LB is one of the most notified vector-borne diseases worldwide with a rising trend in incidence, with over 200,000 new cases of LB diagnosed each year in Europe [5,6]. In Finland, almost 10,000 LB cases were notified in 2024 according to the National Infectious Diseases Register (NIDR), which includes microbiologically confirmed LB cases, and the Register for Primary Health Care Visits (Avohilmo), which includes mainly clinically diagnosed LB cases, maintained by the Finnish Institute for Health and Welfare (THL; https://thl.fi/en) [7].

The actual LB infection rate cannot be concluded from figures of notified incidence, and therefore seroprevalence estimates in population-based surveys provide a good measure of exposure to Bbsl, even if the lack of seroconversion or degradation of antibodies could also cause underestimations. IgG antibodies are detectable in patients in the disseminated phase of LB, and they can persist for at least 20 years [8]. In a global meta-analysis, after eliminating confounding risk factors, the highest confirmed seroprevalence of Bbsl was observed in Europe, with prevalence of 10.3% [9].

Although the notified LB incidence [7] and the abundance of the two Bbsl spreading *Ixodes* tick species have increased in Finland, along with the expansion of ticks into new areas in northern parts of the country [1], our previous studies suggest a decreasing LB seroprevalence trend in the Finnish population [10,11]. In this study, we aimed to further estimate how LB prevalence in the Finnish population has changed from the late 1960s to 2017 by determining Bbsl seroprevalence in population representative samples collected from four different health surveys conducted over the years. Moreover, we evaluated demographic and behavioural factors to Bbsl seropositive status.

## Methods

### Study samples

The total sample set used to determine the Bbsl seroprevalence consisted of 3,990 sera. These samples were collected as part of four different health surveys: Finnish Mobile Clinic Health Survey 1970 (FMC1970), Mini Finland 1980 (MF1980), Health 2000 (Health2000) and FinnHealth 2017 (FH2017) (ca 1,000 samples from each survey). Serum samples from FMC1970 were previously analysed and reported for Bbsl seroprevalence [11] but were re-analysed in this study using the current screening method to ensure comparability across time points. The first survey, FMC1970, was a nationwide cross-sectional health study conducted in 1966–1972 and covered more than 50,000 people aged ≥ 15 years [11,12]. The second survey, MF1980, was carried out in 1978–1980 in 40 study areas around the country focusing on a sample of over 7,000 participants aged ≥ 30 years [12]. The third survey, Health2000, was carried out in 2000–2001, covering around 10,000 participants aged ≥ 18 years living in the mainland Finland [13]. The fourth survey, FH2017, was carried out in 2017, covering more than 10,000 participants aged ≥ 18 years from 50 localities in Finland [14]. These surveys were comprehensive combinations of health interviews and health examinations aiming to represent the Finnish population. Samples were collected from five different university hospital districts: Helsinki (HYKS), Kuopio (KYS), Oulu (OYS), Tampere (TAYS) and Turku (TYKS), representing the geographical area of the residence of the participants (see results).

Data and serum samples were obtained from the biobank administered by the THL. The samples from different health surveys were selected for this study using simple random sampling. Beside serum samples, demographic and other relevant variables were analysed from each of the survey questionnaires. The samples were stored at −20°C.

### First-tier screening assay

All serum samples were screened manually for lgG antibodies with Anti-*Borrelia* plus VlsE ELISA (IgG) (Euroimmun AG, Lübeck, Germany) according to the manufacturer’s instructions. The test is based on whole antigen extracts of the most relevant human pathogenic Bbsl strains and recombinant VIsE protein [15]. The absorbance was measured at 450 nm using Thermo Scientific Multiskan GO microplate spectrophotometer (Thermo Fisher Scientific, Waltham, the United States (US)). The samples were interpreted as positive (lgG result ≥ 22 RU/mL), borderline (lgG result ≥ 16 < 22 RU/mL) or negative (lgG result < 16 RU/mL), as recommended by the manufacturer. An overview of the diagnostic assay algorithm can be seen in Supplementary Figure S1.

### Second-tier confirmatory assay

The samples interpreted as positive or borderline (lgG result ≥ 16) in the Euroimmun IgG ELISA were further analysed with recomBead Borrelia IgG 2.0 (Mikrogen, Neuried, Germany) according to the manufacturer’s instructions. The assay measures reactivity to several *Borrelia* antigens. Briefly, magnetic polystyrene beads (MagPlex beads) coated with 13 different antigens (p100, VlsE, p58, p39, OspA, OspC of *B. burgdorferi* sensu stricto (ss), *B. afzelii* and *B. garinii*, and p18 of *B. burgdorferi* ss, *B. afzelii*, *B. bavariensis*, *B. garinii* and *B. spielmanii*) were used to detect specific IgG antibodies from the serum samples. We used MAGPIX System with Luminex xPONENT software (Mikrogen) and recomQuant evaluation software (Mikrogen) to determine the IgG levels. The serum samples were interpreted as positive (test result ≥ 4 points), borderline (3 points) or negative (0–2 points). The serum samples with a positive or borderline test result were considered Bbsl antibody positive. Antigen-specific antibody binding signal strengths (cut-off index (COI)) were considered separately in the statistical analysis.

### Statistical analysis

The laboratory results were combined with the background data of the participants. Generalised linear models (GLMs) for binomial data, with the logit link function, were used to estimate the association of possible risk factors with Bbsl seropositivity.

Demographic factors included in the analyses were sex, age group, university hospital district, education, employment status and exercise habit of the participant. In addition, some self-reported diseases, symptoms and general health-related questions were selected from the health questionnaires to determine whether Bbsl seropositivity was associated with conditions and symptoms known to be associated with LB. The selected health-related questions comparably presented in each survey questionnaire related to cardiovascular, rheumatic and neurological conditions.

In our final GLM, Bbsl seropositivity was set as a dependent variable, and categorical factors sex, age group, year and university hospital district were included as fixed explanatory effects. Two-way interaction effects were studied among sex, age group and year in the first model. In addition, two-way interactions were studied with a separate model, in which samples from Tampere district were excluded (n = 505), due to missing data in FMC1970, to allow district-related interactions for the rest of the data. Moreover, an explanatory factor, work status, was studied in a separate model, in which only data from FMC1970 and MF1980 were included (n = 1,913), since the missing information from Health2000 and FH2017. When analysing health-related questions, symptoms (binomial yes/no) possibly relating to LB were set as dependent variables and sex, age group, year, university hospital district and Bbsl seropositivity (yes/no) were set as explanatory effects.

Moreover, to assess the impact of different years on the magnitude of serological response to Bbsl antigens (the concentration of IgG antibodies after Euroimmun ELISA and signal strength of IgG antibodies towards VlsE and p18 of *B. afzelii* in the confirmatory recomBead immunoassay), a one-way GLM was conducted with heterogenous variances among years (to meet the assumption of linear models), a lognormal error distribution and an identity link function.

The results (mean probabilities of being seropositive in different factor categories or mean signal strengths in different years) were displayed using model-derived least squares means (i.e. estimated marginal means) with 95% confidence intervals (CI), and the statistical significance level was considered at the 5% level. Probability value (p value) adjustments for multiple comparisons in a posteriori pairwise tests were made with Tukey-Kramer method (details not shown in results). Statistical analyses were carried out using SAS 9.4 via Enterprise Guide 8.4 user interface (SAS Institute Inc., Cary, US).

## Results

We analysed 3,990 serum samples. Detailed information of the samples and background data are presented in Supplementary Table S1. There were slightly more females (n = 2,035) than males (n = 1,955) among the study participants. Age of the participants ranged from 15 to 94 years. However, only nine participants were adolescents (15–17 years old), all from FMC1970. The participants were categorised to five age groups, and each category included around 20% of participants (15–39 years: n = 841; 40–49 years: n = 842; 50–59 years: n = 926; 60–69 years: n = 772), apart from the oldest age group (≥ 70 years), which included 609 (15.3%) participants. Of the samples, 1,105 (27.7%) were collected from Helsinki, 901 (22.6%) from Oulu, 796 (19.9%) from Kuopio, 683 (17.1%) from Turku and 505 (12.7%) from Tampere university hospital district.

### Seropositivity

The first-tier screening assay resulted in 650 (16.3%; 95% CI: 15.1–17.4%) positive and 250 (6.3%; 95% CI: 5.5–7.0%) borderline samples (total of 900 samples; 22.6%; 95% CI: 21.3–23.9%) that were further analysed with the second-tier confirmatory assay. The testing algorithm for the Bbsl seroprevalence is seen in Supplementary Figure S1. After the second-tier confirmatory assay, 479 (53.2%; 95% CI: 50.0–56.5%) samples were positive and 43 (4.8%; 95% CI: 3.4–6.2%) were borderline (total of 522 samples; 58.0%; 95% CI: 54.8–61.2%). Thus, the overall seroprevalence of Bbsl in Finnish adult population during the study period was 13.1% (522/3,990; 95% CI: 12.0–14.1%).

### Factors associated with seropositivity

The factors associated with Bbsl seropositivity in the GLM are shown in Table. Sampling years were significantly associated with seropositivity as a main effect (p < 0.0001) (Table). The probability of a seropositive sample was highest in the samples gathered in 1966–1972 and lower in those gathered in 2000–2001 and 2017. The seroprevalence as a crude percentage was 25.0% in 1966–1972, 16.6% in 1978–1980, 7.4% in 2000–2001 and 3.4% in 2017, as presented in Supplementary Table S1. Moreover, there was a significant difference in the strength of serological responses to Bbsl antigens between the study years as elucidated in Supplementary Figure S2. The highest serological responses were observed in the samples gathered in 1966–1972: in the concentration of antibodies in the first-tier screening assay (GLM: F_3, 896_ = 37.59; p < 0.0001 for the effect of the year), and against VlsE (F_3, 518_ = 6.12; p = 0.0004) and p18 of *B. afzelii* (F_3, 510_ = 4.61; p = 0.0034) in the second-tier confirmatory immunoassay. In the first-tier assay, the concentration of IgG antibodies of the positive samples varied from 16 to > 200, and in the second-tier assay, the signal strength (COI-value) of IgG antibodies towards VlsE and p18 of *B. afzelii* varied between 0 and 10. Most (385/522; 73.8%) of the samples interpreted as positive after the second-tier confirmatory assay had detectable IgG antibody levels (COI ≥ 0.67) towards the p18 of *B. afzelii*, while only a minority of positives contained antibodies towards the p18 of *B. garinii* (n = 12), *B. burgdorferi* ss. (n = 5) or *B. bavariensis* (n = 14).

**Table:** Factors associated with *Borrelia burgdorferi* sensu lato seroprevalence, Finland, 1966–2017 (n = 3,990)^a^

Factor	Least squares mean estimates		GLM: test of fixed effects		
	Mean	95% CI	DF^b^	F value	p value
Sampling years					
1966–1972	0.220	0.187–0.256	3	60.54	< 0.0001
1978–1980	0.109	0.091–0.131			
2000–2001	0.036	0.025–0.050			
2017	0.019	0.012–0.030			
University hospital district					
Helsinki	0.164	0.139–0.192	4	52.47	< 0.0001
Kuopio	0.141	0.116–0.170			
Oulu	0.019	0.014–0.028			
Tampere	0.053	0.035–0.079			
Turku	0.047	0.034–0.063			
Sex					
Female	0.054	0.043–0.067	1	10.16	0.0014
Male	0.081	0.067–0.097			
Age (years)					
15–39	0.025	0.016–0.040	4	22.15	< 0.0001
40–49	0.037	0.025–0.056			
50–59	0.074	0.055–0.099			
60–69	0.103	0.081–0.131			
≥ 70	0.159	0.131–0.193			
Interaction terms					
Sex*Age	Not shown		4	1.01	0.4034
Year*Sex			3	1.82	0.1411
Year*Age			12	2.91	0.0005

CI: confidence interval; Den DF: denominator degrees of freedom; DF: degrees of freedom; F-value (F-statistic): ratio of variances used in hypothesis testing; GLM: generalised linear model.

^a^ Least squares mean estimates (with 95% CI) give the probability of being seropositive according to the conducted generalised linear model.

^b^ Den DF = 3,958.

**Figure 1:**
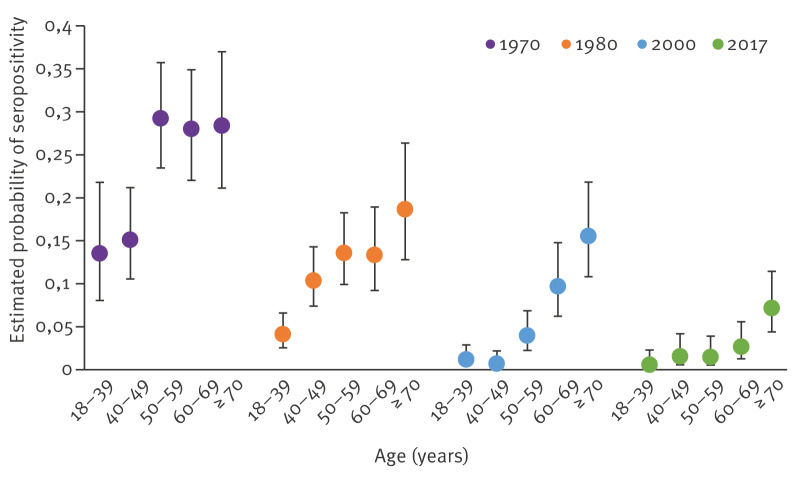
Interaction between sampling year and age group on probability of being *Borrelia burgdorferi* sensu lato seropositive, Finland, 1966–2017 (n = 3,990)^a^

^a^ The interactions were estimated using least squares means with 95% confidence intervals.

University hospital district was significantly associated with seropositivity (p < 0.0001) (Table). The probability of being Bbsl seropositive was higher among residents from southern (Helsinki) or central and eastern Finland (Kuopio) than from western (Turku and Tampere) and northern Finland (Oulu) (Table). As a main effect, males had a significantly higher probability (mean: 0.081; 95% CI: 0.067–0.097) of being seropositive than females (mean: 0.054; 95% CI: 0.043–0.067) (Table). A clear increase in age-related seroprevalence was observed, with the oldest age group (≥ 70 years) having the highest probability of being seropositive (mean: 0.159; 95% CI: 0.131–0.193 (Table). After adjustment for year, sex, age group and university hospital district, main effects of health-related questions were not significantly associated with seropositivity. Health-related questions that were studied are shown in Supplementary Table S1. Furthermore, the final GLM yielded a significant interaction between sampling years and age group (p = 0.0005) (Table, Figure 1). However, the probability of being seropositive seemed to increase with age in each study year (Figure 1). In 1966–1972, all the age groups of ≥ 50 years had higher seroprevalence than the younger age groups (27.9–30.3% vs. 13.4–17.7%, respectively), whereas in the three more recent surveys the oldest age group (≥ 70 years) peaked in seropositivity (Figure 1). The probability generally decreased towards 2017.

Displayed years (1970, 1980, 2000, 2017) correspond to collection periods 1966–1972, 1978–1980, 2000–2001 and 2017.

When analysing the samples from FMC1970 and MF1980 only, the surveys with information on the work status of the participants available, a significant association between seropositivity and the work status was observed (GLM: F_5, 1,913_ = 2.44; p = 0.0326) as shown in Supplementary Table S2. People working outdoors, e.g. in farming, had the highest probability (mean: 0.197; 95% CI: 0.156–0.247) of being seropositive and people working indoors, e.g. office, had the lowest probability (mean: 0.112; 95% CI: 0.081–0.153) of being seropositive.

**Figure 2:**
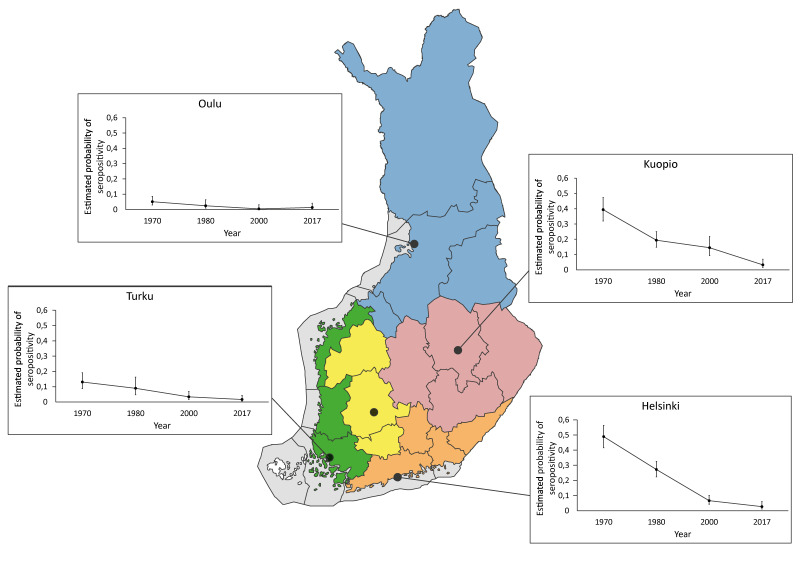
Interaction between sampling years and area of residence on probability of being *Borrelia burgdorferi* sensu lato seropositive, Finland, 1966–2017 (n = 3,990)^a^

^a^ The interactions were estimated using least squares means with 95% confidence intervals.

Because the university hospital district was significantly associated with seropositivity as a main effect (Table), the interaction between sampling years and university hospital district demanded a closer examination. When the 505 samples were removed from Tampere district (missing information from FMC1970), a significant interaction was observed (GLM: F_9, 3,430_ = 2.89, p = 0.0021), as shown in Supplementary Table S3. The differences in seroprevalence among the study areas levelled off in 2017 (Figure 2). The probability of a sample being seropositive decreased significantly in Helsinki and Kuopio university hospital districts between 1966–1972 and 1978–1980 (p < 0.001). The seroprevalence in Helsinki continued to decrease considerably between 1980 and 2000, from 26.3% (95% CI: 21.5–31.1%) to only 8.2% (95% CI: 5.4–11.1%), while at the same time, the decrease in seroprevalence in another high prevalence district, Kuopio, was not prominent (from 19.2%; 95% CI: 14.4–24.0% to 18.8%; 95% CI: 12.7–25.0%).

Displayed years (1970, 1980, 2000, 2017) correspond to collection periods 1966–1972, 1978–1980, 2000–2001 and 2017.

Tampere university hospital district (yellow area) was excluded from the analysis due to missing data in 1970s. Crude prevalence for Tampere district was 0.092 (in 1980), 0.039 (in 2000) and 0.025 (in 2017) as can be deduced from Supplementary Table S1.

Besides in Helsinki district, a significant decrease was observed in Turku district between 1978–1980 and 2000 (p = 0.044). Kuopio was the only district where the probability decreased between 2000–2001 and 2017 (p = 0.001). No significant changes in seropositivity were detected in Oulu district during the study period.

Other examined two-way interactions were non-significant, as seen in Table and Supplementary Table S3, which show the association of factors and Bbsl seropositive status without Tampere district.

## Discussion

We assessed changes in Bbsl seroprevalence over a period from the late 1960s, i.e. the time before the identification of the causative agent of LB, to 2017 when LB was already a widely recognised disease with targeted diagnostic methods for detection and effective antibiotic treatment in place and applied in routine practice. We estimated that Bbsl seroprevalence in Finland in 1966–1972 was 25.0%. Since then, the seroprevalence decreased considerably to 16.6% in 1978–1980, 7.4% in 2000–2001 and 3.4% in 2017. In our previous study, the estimated seroprevalence of Bbsl in Finland in 2011 was 3.9%, which is in accordance with our observed descending seroprevalence curve [10]. A similar decreasing trend in Bbsl seroprevalence has been observed in Czechia between 1978–1989 and 2001 [16], although other studies in European countries indicate either no substantial change or an increase in Bbsl seroprevalence during the last decades [9,17,18]. Nevertheless, there is substantial variation in LB infection rates and Bbsl seroprevalence estimates across the countries, regional areas and particular population groups [9,19], while national representative studies assessing changes in the general population over time remain scarce. The heterogeneity in the studies and LB surveillance systems imposes limitations on comparisons across the countries.

After its recognition in 1975, the notified incidence of LB has increased in several European countries, including Finland, and it is now the most common tick-borne disease in the northern hemisphere [20]. The observed increase in notified LB cases in northern Europe probably reflects the increased abundance and expanded geographic distribution of its vector species, *Ixodes* ticks, and the increased awareness of the LB [21,22]. However, at the same time, the increased awareness among healthcare providers and the general population, as well as the use of antibiotics as an effective treatment method, could be the main reasons for the observed decrease in Bbsl seroprevalence. Although EM and acrodermatitis chronica atrophicans, signs associated also with LB, were treated with penicillin in Europe already in the 1950s, regular antibiotic treatments started in the 1980s [23]. Even though antibodies can remain elevated for years after infection, despite successful treatment [8], the concentration of antibodies declines after antibiotic treatment [24,25]. In our study, the concentrations of IgG antibodies in the positive samples were highest in 1966–1972, whereas the concentration decreased towards the latest study years. Since IgG antibodies towards the p18 protein are associated with late disseminated LB [26], this observation might indicate that 50 years ago people may have had more often an ongoing disseminated LB, or that they had been exposed to Bbsl several times leading to a pronounced immune response, as suggested in our previous study [11]. Interestingly, while *B. garinii* is the most prevalent Bbsl genospecies in Finnish ticks [27], most (73.8%) of our seropositive samples had the IgG antibody level elevated towards the p18 of *B. afzelii*. While the breakthrough in LB diagnostics and treatment in the 1980s and 1990s could be one reason behind the observed decrease in Bbsl seroprevalence, it is likely not the only one, since a major decrease in seroprevalence occurred already between 1966–1972 and 1978–1980, when antibiotics were not yet routinely used for the treatment of LB. Nevertheless, increasing awareness of ticks and related disease risks, especially following the discovery of tick-borne encephalitis (TBE) in Kumlinge, Åland Islands in 1959 [28], may have influenced the decline in seroprevalence in Finland already before the 1980s.

Another underlying cause for decreasing seroprevalence could be the economic and demographic changes in the Finnish society [29]. Finland experienced fast population migration to urban centres, especially during the late 20th century. Fifty years ago, Finland was still largely an agrarian society, relying on agriculture and forestry, and tick-human encounters were likely more frequent than today. When the associations of work status and seropositivity in 1966–1972 and 1978–1980 were analysed, people working outdoors, e.g. in farming and forestry, had the highest probability of being seropositive and people working indoors had the lowest probability of being seropositive. A systematic literature review from Europe has also shown that the seroprevalence estimates are lower in the general population than among risk groups, like forestry workers and hunters [19].

The age-dependent increase in Bbsl seroprevalence was also observed, likely reflecting the cumulative exposure to Bbsl. Moreover, in 1966–1972, seroprevalence was higher among people who were ≥ 50 years old compared with younger age groups, whereas in later years the seroprevalence peaked among people aged ≥ 70 years. Together with the increased awareness and antibiotic treatments, increased life expectancy and improved health among aging people may have reduced the cumulative exposure to Bbsl. As a result, overall seroprevalence has declined, and the peak in seropositivity is now observed among the oldest age group. Previous studies have shown a bimodal age distribution in LB cases [30,31], with peaks in young children and older adults, and also notable Bbsl seroprevalence among children has been observed [32]. Our study is limited in this respect, as it did not include children and included only few adolescents (15–17 years old). Males had also a higher probability of being seropositive than females. The higher seroprevalence in males and in older age groups has been seen in other studies as well [10,11,33,34]. Interestingly, most European studies show higher seroprevalence in males, but LB incidence is often higher in females [7,35]. The contradiction between seroprevalence and incidence rates in Europe could be due to gender-related behavioural differences in healthcare seeking or genetic variations in immune system.

We compared seroprevalence estimates of five university hospital districts and found regional variation. The probability of being Bbsl seropositive was higher among residents from southern (Helsinki) or central and eastern Finland (Kuopio) than from western (Turku and Tampere) and northern Finland (Oulu). Residents from northern Finland had the lowest seroprevalence rate, as expected due to lower tick abundance in the north. Surprisingly, the seroprevalence was quite low in south-west Finland as well, which today has one of the highest tick densities in Finland [36,37]. Observed differences might be caused by varying levels of awareness about ticks and LB among laymen and physicians, since south-west Finland has long had one of the highest incidences of LB and TBE [7,28]. Another interesting result was that the seroprevalence in Helsinki district decreased considerably between 1980 and 2000, from 26.3% to only 8.2%, while at the same time, the decrease in seroprevalence in another high prevalence district, Kuopio, was quite modest (from 19.2% to 18.8%). The seroprevalence in northern Finland was very low just over a decade ago, being 0.67% in 2000 and 0.87% in 2011 [10]. Seroprevalence in northern Finland may have increased, being 2.0% in 2017, which may reflect the increasing tick, especially *I. persulcatus*, abundance in northern Finland in recent decades [27]. From 1995 to 2014, the notified incidence of LB has increased most significantly in western, southern and south-eastern Finland [7]. Nevertheless, Bbsl seroprevalence may vary substantially even within a university hospital district.

Bbsl seropositivity was not statistically associated with any of the health-related background information after adjusting for demographic factors. Our previous study noticed that in 1970 in Finland, self-reported perception of feeling unhealthy, previous heart failure and heart valvular disease were associated with Bbsl seropositivity [11]. One of the reasons for not finding significant correlations with any health-related questions might be the small number of seropositive subjects in these categories. Nevertheless, our results are in accordance with findings from Norway, where no subjective health complaints were associated with Bbsl seropositivity [38].

The reason we re-analysed the samples from 1970, which were analysed for IgG seroprevalence in our previous study using a whole-cell sonicate IgG ELISA, C6 peptide ELISA and recomBead IgG 2.0 [11], was to get a more comprehensive view of the seroprevalence changes in Finland and to get comparable results. Our present study, using a different analytical method, resulted in the seroprevalence of 25% in 1966–1972, which was higher than our previous result (20%). All positive samples from our previous study were also positive in the current study, but an additional 49 samples were positive in the re-analysis. This highlights how serological results vary depending on the method used, complicating comparisons across studies. A two-tier system used in our study, which utilises several Bbsl antigens, has a high sensitivity and specificity, > 90% [39]. Nevertheless, the severity and length of infection or cross-reactions with other microbes might still affect the results. Antibodies might also degrade during the storage over a long-period of time, even if storing antibodies at −20°C should be adequate. However, samples from FMC1970 were quality-checked by measuring antibody levels towards varicella zoster virus (VZV) in the previous study [11]. Moreover, our results showing a considerably higher seroprevalence in the early sampling periods suggests that antibody degradation during storage is unlikely to be a major source of bias in our results, although some degree of degradation cannot be excluded.

## Conclusion

In conclusion, our study reveals a decreasing trend in seroprevalence among the Finnish adult population in the past 50 years. Moreover, our results strengthen the previously observed findings of higher Bbsl seroprevalence in males and in older age groups. We demonstrate how repeated serosurveys over a period of time can be helpful in revealing long-term trends and in identifying risk groups. Further studies, for example temporal studies within different risk groups or case-control studies, are still needed to shed light on the underlying causes for the observed decline in Bbsl seroprevalence in Finland.

## Data Availability

The data will be made available by the corresponding author upon reasonable request. Access may be subject to institutional or ethical approvals, depending on the nature of the request.

## Supplementary Data

493000 Supplementary Material
