# Supplementary material for: Repeated cross-sectional surveys show a decreasing trend in Borrelia burgdorferi sensu lato seroprevalence over a 50-year period, Finland, 1966 to 2017
Source: Euro Surveill. 2025 Sep 11;30(36):2500171. doi: 10.2807/1560-7917.ES.2025.30.36.2500171 (PMC12432490; doi:10.2807/1560-7917.ES.2025.30.36.2500171)
Supplement: Supplementary Material [file 25-00171_LAMPPU_Supplement.pdf]

Supplementary Material

"This supplementary material is hosted by Eurosurveillance as supporting information alongside the article [Decreasing trend in seroprevalence of Borrelia burgdorferi sensu lato and the associated risk factors in Finland over a 50-year period], on behalf of the authors, who remain responsible for the accuracy and appropriateness of the content. The same standards for ethics, copyright, attributions and permissions as for the article apply. Supplements are not edited by Eurosurveillance and the journal is not responsible for the maintenance of any links or email addresses provided therein."

Figure S1: Schematic overview of diagnostic assay algorithm and results

Figure S2: Serological responses to *Borrelia* antigens in the positive samples among study years.

Table S1: Background data with sample sizes, factors of statistical analyses and Bbsl seroprevalences

Table S2: Results from GLM with work status as a factor

Table S3: Results from GLM without Tampere university hospital district

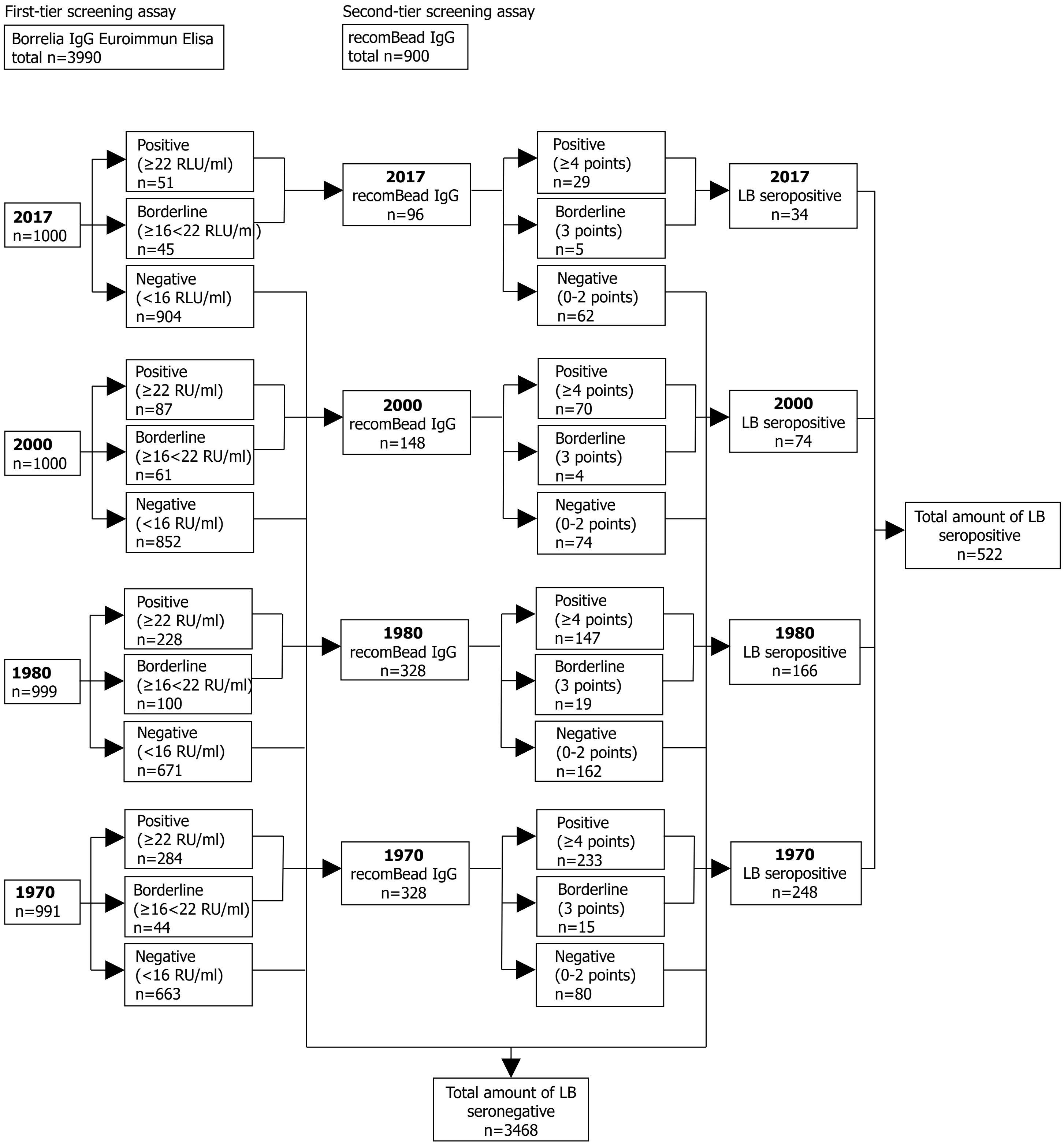

Supplementary Figure S1. Schematic overview of diagnostic assay algorithm and results.

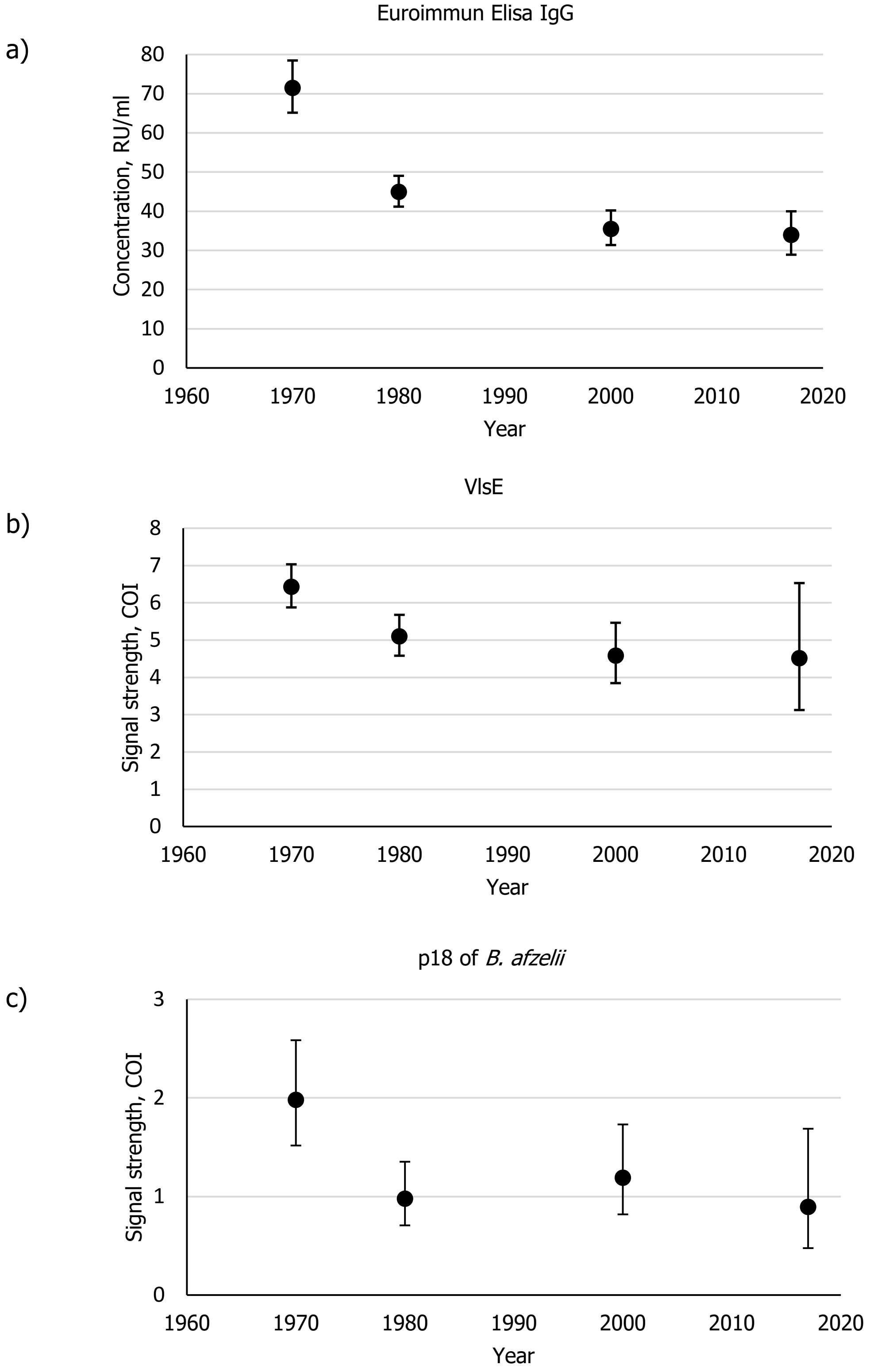

Supplementary Figure S2. Serological responses to *Borrelia* antigens in the positive samples among study years. a) The concentration of the IgG antibodies in the positive samples after the screening Euroimmun Elisa assay, b) the signal strength of IgG antibodies toward VlsE and c) p18 of *B. afzelii* after the confirmatory recomBead immunoassay. Data represent back-transformed least-squares means (with asymmetric 95 % confidence intervals) from GLMs with lognormal error distributions.

Supplementary Table S1. Background data with sample sizes, factors of statistical analyses and Bbsl seroprevalences

			**Total**		**1970**		**1980**		**2000**		**2017**	
**Factor**	**No. persons analysed**	**percent (%)**	No. persons IgG positive	Crude Percent	No. persons IgG positive	Crude Percent	No. persons IgG positive	Crude Percent	No. persons IgG positive	Crude Percent	No. persons IgG positive	Crude Percent
TOTAL	3990	100	522/3990	13.08	248/991	25.03	166/999	16.62	74/1000	7.40	34/1000	3.40
**Area**												
Helsinki	1105/3990	27.69	240/1105	21.72	117/226	51.77	86/327	26.30	29/352	8.24	8/200	4.00
Kuopio	796/3990	19.27	162/796	20.35	73/182	40.11	50/260	19.23	29/154	18.83	10/200	5.00
Turku	683/3990	17.12	52/683	7.61	25/181	13.81	11/110	10.00	9/192	4.69	7/200	3.50
Tampere	505/3990	12.66	25/505	4.95			14/152	9.21	6/153	3.92	5/200	2.50
Oulu	901/3990	22.58	43/901	4.77	33/402	8.21	5/150	3.33	1/149	0.67	4/200	2.00
**Sex**												
Female	2035/3990	51.00	228/2035	11.20	108/447	24.16	73/515	14.17	33/551	5.99	14/522	2.68
Male	1955/3990	49.00	294/1955	15.04	140/544	25.74	93/484	19.22	41/449	9.13	20/478	4.18
**Age (years):**												
15-39	841/3990	21.08	40/841	4.76	15/112	13.39	18/257	17.00	5/240	2.08	2/232	0.86
40-49	842/3990	21.10	82/842	9.74	33/186	17.74	42/257	16.34	3/233	1.29	4/166	2.41
50-59	926/3990	23.21	145/926	15.66	84/290	28.97	44/223	19.73	13/212	6.13	4/201	1.99
60-69	772/3990	19.35	130/772	16.84	70/251	27.89	32/148	21.62	21/161	13.04	7/212	3.30
>70	609/3990	15.26	125/609	20.53	46/152	30.26	30/114	26.32	32/154	20.78	17/189	8.99
**Education:**												
Lower lever	1465/2987	49.05	211/1465	14.40	NA	NA	162/918	17.65	41/383	10.70	8/164	4.88
Middle level	704/2987	23.57	36/704	5.11	NA	NA	0/38	0	22/321	6.85	14/345	4.06
Higher level	818/2987	27.39	27/818	3.30	NA	NA	4/43	9.30	11/292	3.77	12/483	2.48
**Health**												
Moderate or good	2362/2972	79.48	294/2362	12.45	126/603	20.90	135/826	16.34	NA	NA	33/933	3.54
Poor	610/2972	20.52	146/610	23.93	114/373	30.56	31/173	17.92	NA	NA	1/64	1.56
**Current employment status:**												
Employed	1703/1907	89.30	139/1703	8.16	131/610	21.48	NA	NA	18/571	3.15	10/522	1.92
Unemployed	204/1907	10.70	22/204	10.78	21/70	30.00	NA	NA	0/73	0	1/61	1.64
**Sports/exercise activities:**												
No	552/3444	16.03	79/552	14.31	NA	NA	61/349	17.48	13/102	12.75	5/101	4.95
Occasionally	826/3444	23.98	126/826	15.25	27/97	27.84	90/513	17.54	6/109	5.50	3/107	2.80
Regularly	2066/3444	59.99	226/2066	10.94	132/491	26.88	15/137	10.95	55/767	7.17	24/671	3.58
**Work**												
Indoor, office etc.	365/1938	18.83	48/365	13.15	27/162	16.67	21/203	10.34	NA	NA	NA	NA
Industrial, transportation etc.	562/1938	29.00	127/562	22.60	89/332	26.81	38/230	16.52	NA	NA	NA	NA
Outdoor, farming etc.	413/1938	21.31	105/413	25.42	75/282	26.60	30/131	22.90	NA	NA	NA	NA
Pensioner	274/1938	14.14	56/274	20.44			56/274	20.44	NA	NA	NA	NA
Service	174/1938	8.98	31/174	17.82	19/76	25.00	12/98	12.24	NA	NA	NA	NA
Stay at home parent	150/1938	7.74	30/150	20.00	21/87	24.14	9/63	14.29	NA	NA	NA	NA

**Health-related questions**												
**Depression**												
no	2532/2931	86.39	218/2532	8.61	NA	NA	136/829	16.41	52/786	6.62	30/917	3.27
yes	399/2931	13.61	50/399	12.53	NA	NA	26/148	17.57	20/188	10.64	4/63	6.35
**Chronic disease**												
no	1532/2970	51.58	116/1532	7.57	NA	NA	81/548	14.78	20/506	3.95	15/478	3.14
yes	1438/2970	48.42	156/1438	10.85	NA	NA	85/451	18.85	54/491	11.00	17/496	3.43
**Limited moving**												
no	1290/1978	65.22	127/1290	9.85	NA	NA	107/686	15.60	NA	NA	20/604	3.31
yes	688/1978	34.78	73/688	10.61	NA	NA	59/313	18.85	NA	NA	14/375	3.73
**Sleeping problems**												
no	1791/2939	60.94	103/1791	5.75	NA	NA	29/202	14.36	42/653	6.43	32/936	3.42
yes	1148/2939	39.06	166/1148	14.46	NA	NA	133/775	17.16	31/327	9.48	2/46	4.35
**Headache**												
no	1354/1951	69.40	164/1354	12.11	NA	NA	109/651	16.74	55/703	7.82	NA	NA
yes	597/1951	30.60	70/597	11.73	NA	NA	53/324	16.36	17/273	6.23	NA	NA
**Dizziness**												
no	1439/1941	74.14	160/1439	11.12	NA	NA	115/690	16.67	45/749	6.01	NA	NA
yes	502/1941	25.86	76/502	15.14			47/281	16.73	29/221	13.12	NA	NA
**Hot and cold**												
no	1612/1945	82.88	193/1612	11.97	NA	NA	132/769	17.17	61/843	7.24	NA	NA
yes	333/1945	17.12	42/333	12.61	NA	NA	30/204	14.71	12/129	9.30	NA	NA
**Tingling**												
no	778/1953	39.84	88/778	11.31	NA	NA	61/376	16.22	27/402	6.72	NA	NA
yes	1175/1953	60.16	147/1175	12.51	NA	NA	101/598	16.89	46/577	7.97	NA	NA
**Continuous pains**												
no	1391/1946	71.48	145/1391	10.42	NA	NA	105/661	15.89	40/730	5.48	NA	NA
yes	555/1946	28.52	89/555	16.04	NA	NA	57/311	18.33	32/244	13.11	NA	NA
**Tiredness**												
no	1363/1976	68.98	149/1363	10.93	NA	NA	110/689	15.97	39/674	5.79	NA	NA
yes	613/1976	31.02	90/613	14.68	NA	NA	56/310	18.06	34/303	11.22	NA	NA
**Weakened memory**												
no	1723/1956	88.09	197/1723	11.43	NA	NA	131/800	16.38	66/923	7.15	NA	NA
yes	233/1956	11.91	39/233	16.74	NA	NA	31/177	17.51	8/56	14.29	NA	NA

Supplementary Table S2. Association of risk factors and Borrelia burgdorferi seropositive status in Finland in 1970 and 1980, including the work status. Least-squares mean estimates (with 95% confidence limits) give the probability of being seropositive according to the conducted GLM.

**Factor**	**Least-squares mean estimates**			**GLM: Test of fixed effects**			
	Mean	Lower 95% CL	Upper 95% CL	Num DF	Den DF	F- value	*p*-value
**Work status**				5	1913	2.44	0.0326
Indoor, office etc.	0.1118	0.0807	0.1530				
Industrial, transportation etc.	0.1699	0.1343	0.2125				
Outdoor, farming etc.	0.1973	0.1556	0.2470				
Pensioner	0.1174	0.0749	0.1794				
Service	0.1731	0.0340	0.2502				
Stay at home parent	0.1496	0.0317	0.2228				
**Year**				1	1913	14.29	0.0002
1970	0.1991	0.1611	0.2435				
1980	0.1122	0.0897	0.1395				
**University hospital district**				4	1913	44.48	<.0001
Helsinki	0.3643	0.3176	0.4138				
Kuopio	0.2717	0.2275	0.3208				
Turku	0.0992	0.0701	0.1386				
Tampere	0.1284	0.0760	0.2086				
Oulu	0.0085	0.0339	0.0678				
**Sex**				1	1913	1.44	0.2299
Female	0.1395	0.1150	0.1683				
Male	0.1623	0.1316	0.1986				
**Age**				4	1913	8.25	<.0001
15-39	0.0727	0.0489	0.1067				
40-49	0.1150	0.0862	0.1519				
50-59	0.1888	0.1518	0.2324				
60-69	0.1975	0.1533	0.2506				
>70	0.2308	0.1719	0.3025				
**Sex*Age**				4	1913	0.98	0.4182
**Year*Sex**				1	1913	1.07	0.3006
**Year*Age**				4	1913	1.71	0.1446

Supplementary Table S3. Association of risk factors and Borrelia burgdorferi seropositive status in Finland without Tampere university hospital district. Least-squares mean estimates (with 95% confidence limits) give the probability of being seropositive according to the conducted GLM.

Factor	Least-squares mean estimates			GLM: Test of fixed effects			
	Mean	Lower 95% CL	Upper 95% CL	Num DF	Den DF	F- value	*p*-value
**Year**				3	3430	32.18	<.0001
1970	0.211	0.177	0.249				
1980	0.109	0.081	0.145				
2000	0.035	0.020	0.063				
2017	0.021	0.012	0.037				
**University hospital district**				3	3430	20.13	<.0001
Helsinki	0.140	0.111	0.176				
Kuopio	0.148	0.118	0.184				
Turku	0.051	0.035	0.075				
Oulu	0.016	0.006	0.033				
**Sex**				1	3430	6.14	0.0133
Female	0.055	0.041	0.073				
Male	0.080	0.062	0.103				
**Age**				4	3430	11.19	<.0001
15-39	0.020	0.009	0.042				
40-49	0.046	0.029	0.072				
50-59	0.077	0.051	0.113				
60-69	0.104	0.077	0.140				
>70	0.160	0.121	0.208				
**Sex*Age**				4	3430	0.89	0.4679
**Year*Sex**				3	3430	1.11	0.3423
**Year*Age**				12	3430	2.74	0.0011
**District*Year**				9	3430	2.89	0.0021
**District*Sex**				3	3430	0.37	0.7735
**District*Age**				12	3430	1.40	0.1574
